# CRISPR-mediated ablation of overexpressed EGFR in combination with sunitinib significantly suppresses renal cell carcinoma proliferation

**DOI:** 10.1371/journal.pone.0232985

**Published:** 2020-05-15

**Authors:** Bin Liu, Olivia Adaly Diaz Arguello, Deng Chen, Siwei Chen, Ali Saber, Hidde J. Haisma

**Affiliations:** Department of Chemical and Pharmaceutical Biology, Groningen Research Institute of Pharmacy, Groningen, University of Groningen, Groningen, The Netherlands; Hirosaki University Graduate School of Medicine, JAPAN

## Abstract

Receptor tyrosine kinases, such as VEGFR, PDGFR and EGFR, play important roles in renal cancer. In this study, we investigated *EGFR* knockout as a therapeutic approach in renal cell carcinoma (RCC). We showed that a renal cell carcinoma cell line (RC21) has higher expression of EGFR as compared to other frequently used cell lines such as HEK293, A549, Hela and DLD1. Ablation of *EGFR* by CRISPR/Cas9 significantly restrained tumor cell growth and activated the MAPK (pERK1/2) pathway. The VEGFR and PDGFR inhibitor, sunitinib, attenuated the expression of MAPK (pERK1/2) and pAKT induced by EGFR loss and further inhibited *EGFR*^*-/-*^ cell proliferation. We showed that loss of EGFR eventually leads to resistance to SAHA and cisplatin. Furthermore, EGFR loss induced G2/M phase arrest and resulted in an increased resistance to TNF-related apoptosis-inducing ligand (TRAIL) in renal cell carcinoma. Thus, ablation of overexpressed EGFR by CRISPR/Cas9 alone or in combination with sunitinib may be a new treatment option for renal cell carcinoma.

## Introduction

RCC is one of the most aggressive malignant tumors, accounting for 3% of adult malignancies in Europe and the United States [1]. The 5-year survival rate of metastatic RCC is less than 10% [2]. Treatment options for RCC are limited due to multi-drug resistance including chemotherapy and radiation resistance [3]. Given that RCC is a highly aggressive with poor prognosis cancer, more intensive studies on tumorigenesis and new treatment strategies are required.

The epidermal growth factor receptor (EGFR), vascular endothelial growth factor receptor (VEGFR) and platelet-derived growth factor receptor (PDGFR) play significant roles in RCC progression. Multi-targeted (receptor) tyrosine kinase inhibitors such as sunitinib and sorafenib are commonly used to treat patients with RCC. These TKIs act via blocking VEGFR and/or PDGFR-β in tumor cells. However, more than 30% of patients with RCC who are treated with sunitinib or sorafenib develop hypertension, of whom approximately 12% with a grade 3 hypertension [4]. Combination therapy is another treatment option in which patients are administered with a mixture of different tyrosine kinase inhibitors (TKIs) to get a higher response rate. Several phase III clinical trials (NCT02231749, NCT02420821 and NCT01582672) are currently in process on such therapies. However, a phase II clinical trial reported that sunitinib in combination with gefitinib (an EGFR-TKI) had comparable efficacy to sunitinib as monotherapy [5].

Although crosstalk between EGFR, PDGFR and VEGFR is complicated, two key downstream pathways are shared between them; i.e. the PI3K/AKT and RAS/RAF/MEK/ERK oncogenic pathways [6,7]. These two key pathways are common therapeutic targets for cancer therapy. In this study, we investigated *EGFR* knockout as a therapeutic option in RCC using CRISPR/Cas9 [8–10]. We also evaluated the inhibitory effects of multiple inhibitors as well as alterations in PI3K/AKT and RAS/RAF/MEK/ERK downstream pathways in the *EGFR*^*wt/wt*^ and *EGFR*^*-/-*^ renal cancer cells.

## Materials and methods

### Cell lines

HEK293 (human embryonic kidney), Hela (cervical cancer), A549 (non-small cell lung carcinoma) and DLD1 (colorectal adenocarcinoma) cells were purchased from ATCC. HEK293 (human embryonic kidney) and Hela (cervical cancer), were cultured in DMEM containing 10% fetal bovine serum (FBS) and 1% penicillin/streptomycin. The renal carcinoma cell line RC21 was described elsewhere [11]. RC21, A549 and DLD1 were cultured in RPMI-1640 with 10% FBS and 1% penicillin/streptomycin. Cells were cultured under a humidified 5% Carbon dioxide (CO2) atmosphere at 37°C.

### Generating the RC21 EGFR knockout cell line using CRISPR/Cas9

Generating gene knockout cell line has been described previously [10]. Briefly, The guide RNAs (gRNAs) were derived from the GeCKO (v2) library (Table 1). EGFR CRISPR/Cas9 KO Plasmid (human) consists of a pool of three plasmids, each encoding the Cas9 nuclease and a target-specific 20-nucleotide gRNA designed for maximum knockout efficiency. For transfection, 3 × 10^5^ cells per well were seeded in a 6-well plate. CRISPR/Cas9 plasmids were co-transfected with HDR plasmids which carried the puromycin resistance gene using Lipofectamine 3000 (Invitrogen, Carlsbad, USA). To pick up single clones, 1000 cells were seeded in a 10 cm dish after transfection and puromycin selection for 72 hrs. After two weeks, the culture medium was carefully removed and the dish was rinsed with PBS twice to remove floating cells. Sterile cloning cylinders were placed over each colony. Then, 100 μL of 0.25% trypsin was added to each cylinder, followed by 5 min incubation at 37 °C. Next, 200 μL of medium was added into each cylinder, mixed and the mixtures were transferred to a 6-well plate pre-filled with 2 mL culture medium in each well. *EGFR* knockout clones further validated by Sanger sequencing and western blot.

**Table 1:** List of gRNA sequences for EGFR.

Name	Strand	Sequence
gRNA-1	F	5’-TGAGCTTGTTACTCGTGCCT-3’
	R	5’-AGGCACGAGTAACAAGCTCA-3’
gRNA-2	F	5’-GAGTAACAAGCTCACGCAGT-3’
	R	5’-ACTGCGTGAGCTTGTTACTC-3’

### T7 endonuclease I assay to detect CRISPR/Cas9 induced mutations

Hek293 cells were harvested and genomic DNA was isolated using the (Qiagen, Germany) following manufacturer’s instructions. The concentration of the isolated genomic DNA was determined using The NanoDrop One Spectrophotometer (ThermoFisher Scientific, USA). Then a PCR was performed using Taq polymerase (NEB, USA) with primers in Table 2 for amplification (Sigma-Aldrich, Germany). The PCR amplification was as following an initial denaturation 95 °C for 5 mins, samples were subjected to 35 cycles of 30 denaturation at 95 °C, annealing at 53 °C for 30 seconds followed by extension at 72 °C for 40 seconds. Amplified DNA products were mixed with 1,5μl NEBuffer 2 and 3,0μl nuclease free water. An initial denaturation was performed following a ramp rate -2 °C /second from 95 °C and then -0.1 °C/second from 85 °C to 25 °C, subsequently, 1μl T7e1 enzyme (NEB, USA) was added and incubated at 37 °C in a water bath for 15 mins. Gel electrophoresis was performed for detecting of DNA fragments.

**Table 2:** List of PCR primers for detecting gene knockout/knock-in.

Name	Strand	Sequence
KO exon 2	F	5’- TGGACCTTGAGGGATTGTTT-3’
	R	5’- CCAGATTAGCCTGTTTCTATTTGAT-3’

### Antibodies and chemical reagents

The primary antibodies MAPK (Erk) (#9102, 1:1000), Akt (1:1000, #9272), Phospho-EGF Receptor (Tyr1068) (1:1000, #2234), p(Thr308)-Akt (1:1000, #9275), Phospho-Akt (Ser473) (1:1000, #9271), Phospho-MAPK (pERK) (1:1000, #9101), β-Actin (1:10000, #4967) were purchased from Cell Signaling (Leiden, The Netherlands) and anti-EGFR (1:1000, sc-03-G) was purchased from Santa Cruz Biotechnology(Texas, USA). Cetuximab (ERBITUX) was ordered from Merck (Dietikon, Switzerland). Gefitinib (Iressa) was bought from Sigma (Zwijndrecht, The Netherlands); sunitinib was purchased from LC Laboratories (Woburn, USA). Entinostat and SAHA were purchased from Selleckchem (Munich, Germany). Staurosporine and cisplatin were purchased from Sigma-Aldrich (Zwijndrecht, Nederland). Doxorubicin was purchased from Teva Pharmaceuticals. All drugs were aliquoted in DMSO and stored at -20°C. The human epidermal growth factor (hEGF) and platelet-derived growth factor (PDGF) were purchased from Sigma-Aldrich (Zwijndrecht, Nederland).

### Immunoblotting

Cells were lysed using ELB-softer buffer (50mM Hepes pH7.5, 150mM NaCl, 5mM EDTA, 0,1% NP-40) with PhosSTOP Phosphatase Inhibitor Cocktail (Roche, Mannheim, Germany) and a protease Inhibitor Cocktail (Thermo Fisher Scientific, Waltham, USA). Protein concentration was determined by a standard protocol according to the Pierce BCA Protein Assay Kit (Thermo Fisher Scientific, USA). Twenty micrograms of each sample was loaded and separated by pre-cast SDS-PAGE (Bio-Rad, Hercules, USA) and transferred into a PVDF (polyvinylidene difluoride) membrane. The membrane was incubated with blocking buffer containing 5% skimmed milk with 0.1% (v/v) Tween 20 in 1x PBS (PBST) at room temperature (RT) for 1 hr. Then, the membrane was incubated overnight with the primary antibody at 4°C, followed by the secondary antibody treatment at RT for 1 hr. Bands were imaged using Western Lightning Plus-ECL kit (PerkinElmer, Waltham, USA) and were analyzed by GeneSnap image software (SynGene, Frederick, USA).

### Flow cytometric analysis of EGFR membrane expression

Cells were washed twice with PBS and harvested by trypsin for 5 mins, followed by 1 hr incubation with primary antibody or IgG isotype control on ice. Then, cells were washed by FACS buffer (2% calf serum in PBS) three times and incubated with secondary antibody for 1 hr. Next, cells were washed with FACS buffer three times and protein expression was determined by FACSCalibur flow cytometer (BD, Franklin Lakes, USA).

### Growth inhibition assay

A total of 3x10^3^ cells per well were seeded in 96-well plates and cultured overnight. Afterwards, cells were separately treated with different drugs for 3 days. Next, cells were incubated with medium containing MTS reagent for 90 mins at 37°C according to the instruction from CellTiter 96 AQueous One Solution (Promega, Madison, USA). The absorbance was determined using a Synergy H1 plate reader (BioTek, Winooski, USA) at wavelength of 490 nm. Experiments were performed in triplicates and repeated for three times.

### Clonogenic assay

Clonogenic assay has been described elsewhere [12]. Briefly, A total number of 20,000 cells per well were seeded in a 12-well plate and cultured for 6. Then, the medium was gently removed. Cells were washed with PBS, and then cells were fixed by using 4% formaldehyde. Cells were stained with 1% crystal violet for 20 mins and washed thoroughly by PBS before colonies were counted. For quantification, 0.5 ml 10% acetic acid per well was used to extract the dye. The absorbance was detected at wavelength of 590 nm using a Synergy H1 plate reader (BioTek, Winooski, USA). Experiments were performed in triplicate and repeated at least three times.

### TRAIL protein production

The production and purification of TRAIL protein have been described previously [13]. Briefly, recombinant human sTRAIL protein was produced by *E*. *coli* BL21(DE3) in 2YT medium with 100μg/mL ampicillin and 1% (w/v) glycerol at 37°C to mid-log phase. The protein production was induced by IPTG (0.1mM) and ZnSO_4_ was added to help stabilize the trimer formation. The cells were grown at 20°C overnight after induction. The concentrated pellet was disrupted by sonication. The purification was performed through cation exchange chromatography and gel filtration.

### Apoptosis assay and cell cycle analysis

A total of 5x10^5^ cells per well were seeded in 6-well plates and cultured overnight. The cells were treated with drugs for 24 hrs. Apoptosis and cell cycle were investigated using the eBioscience™ Annexin V Apoptosis Detection Kit APC according to the manufacturer’s protocol (Thermo Fisher Scientific, Waltham, USA) by FACS Calibur flow cytometer (BD, Franklin Lakes, USA). The FACS data were analyzed using FLOWJO v10.1.

## Results

### EGFR is highly expressed in renal cell carcinoma

RC21 is a renal cell carcinoma cell line with overexpressed EGFR [11]. We used flow cytometry to compare the EGFR expression levels in RC21 and four other commonly used cell lines (HEK293, Hela, A549 and DLD1). We showed that the expression level of EGFR in RC21 is 13-fold higher than HEK293, 7-fold higher than DLD1, 5-fold higher than Hela and 4-fold higher than A549 cells (S1 Fig).

### Generating *EGFR* gene knockout cell lines using CRISPR/Cas9

**Fig 1:**
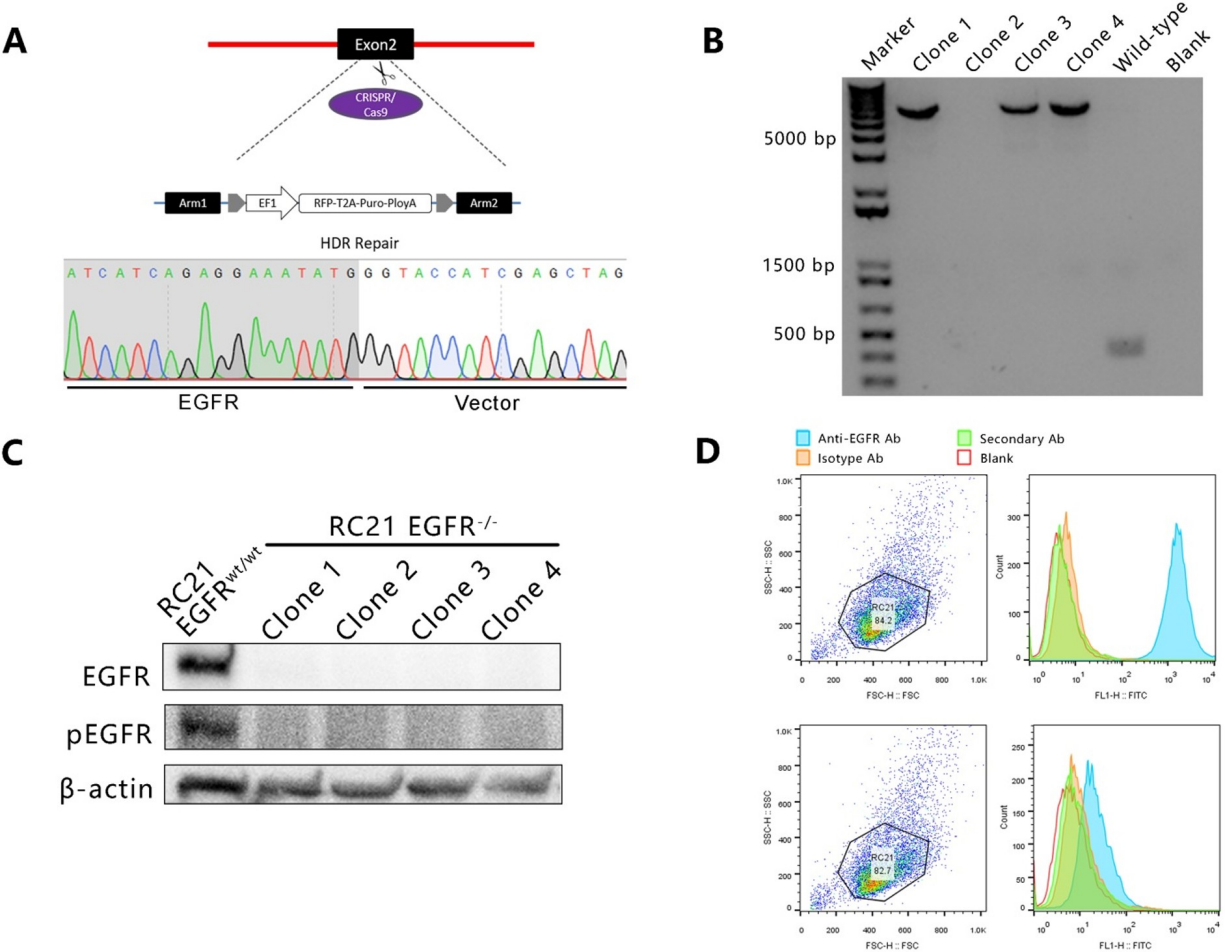
(A) CRISPR-mediated EGFR knockout and homology directed repair (HDR). (B) Agarose gel electrophoresis of PCR-based genotyping for identification of EGFR knockout clones. (C) Western blot analysis of RC21 EGFRwt/wt and EGFR-/- cells. (D) Flow cytometric analysis shows EGFR expression in RC21 EGFRwt/wt and EGFR-/- cells.

To generate a RC21 *EGFR* knockout cell line, a CRISPR/Cas9 approach was employed using two gRNAs targeting exon 2 of *EGFR* with the homology-directed DNA repair (HDR) templates specific to the cut sites of EGFR (Fig 1A). The knockout efficiency was pre-assessed in HEK293 cell line using T7 Endonuclease 1 (T7E1) assay. The indel frequencies induced by CRISPR/Cas9 were up to 60% after sorting for eGFP-positive cells by flow cytometry (S2 Fig). The gRNA/Cas9 and HDR donor plasmids pool were co-transfected to RC21 cells. Four independent *EGFR* knockout clones were picked and expanded and PCR results showed successful disruption of *EGFR* exon 2 by insertion of a donor DNA fragment which was further confirmed by Sanger sequencing (Fig 1A and 1B). Ablation of EGFR was also validated by Western blot and flow cytometry (Fig 1C and 1D).

### EGFR loss inhibits cell proliferation, but leads to resistance to cisplatin and SAHA in renal cell carcinoma

**Fig 2:**
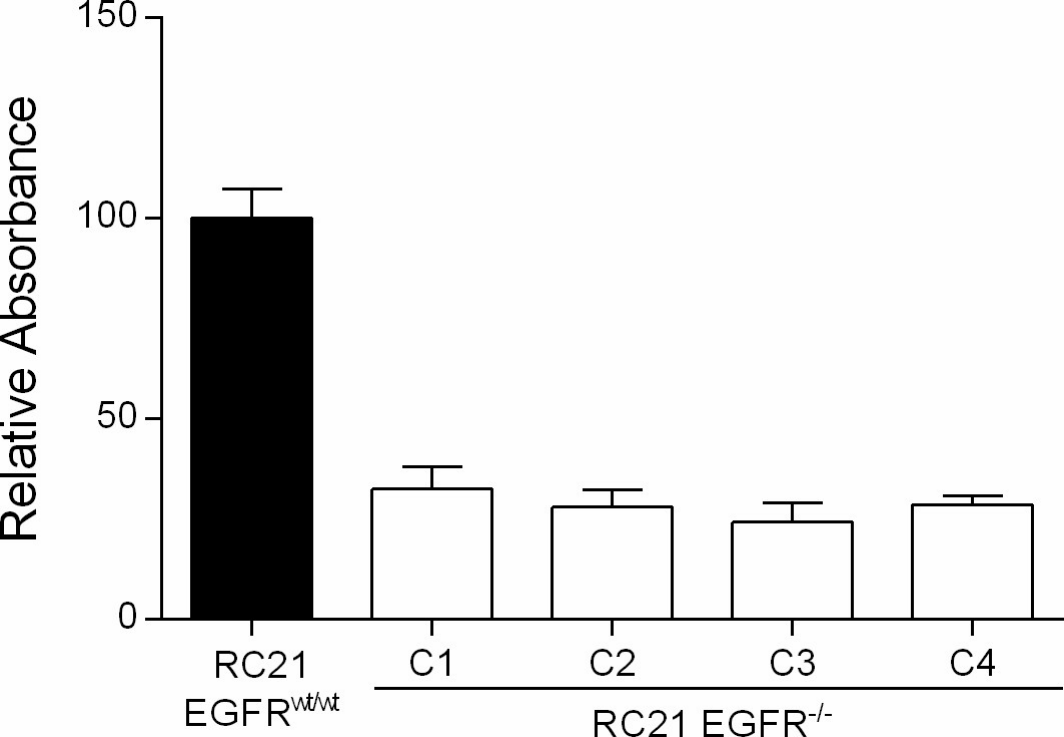
Colony formation assay of RC21 *EGFR*^*wt/wt*^ and *EGFR*^*-/-*^ cells to determine cell proliferation ability of four independent *EGFR* knockout clones.

**Fig 3:**
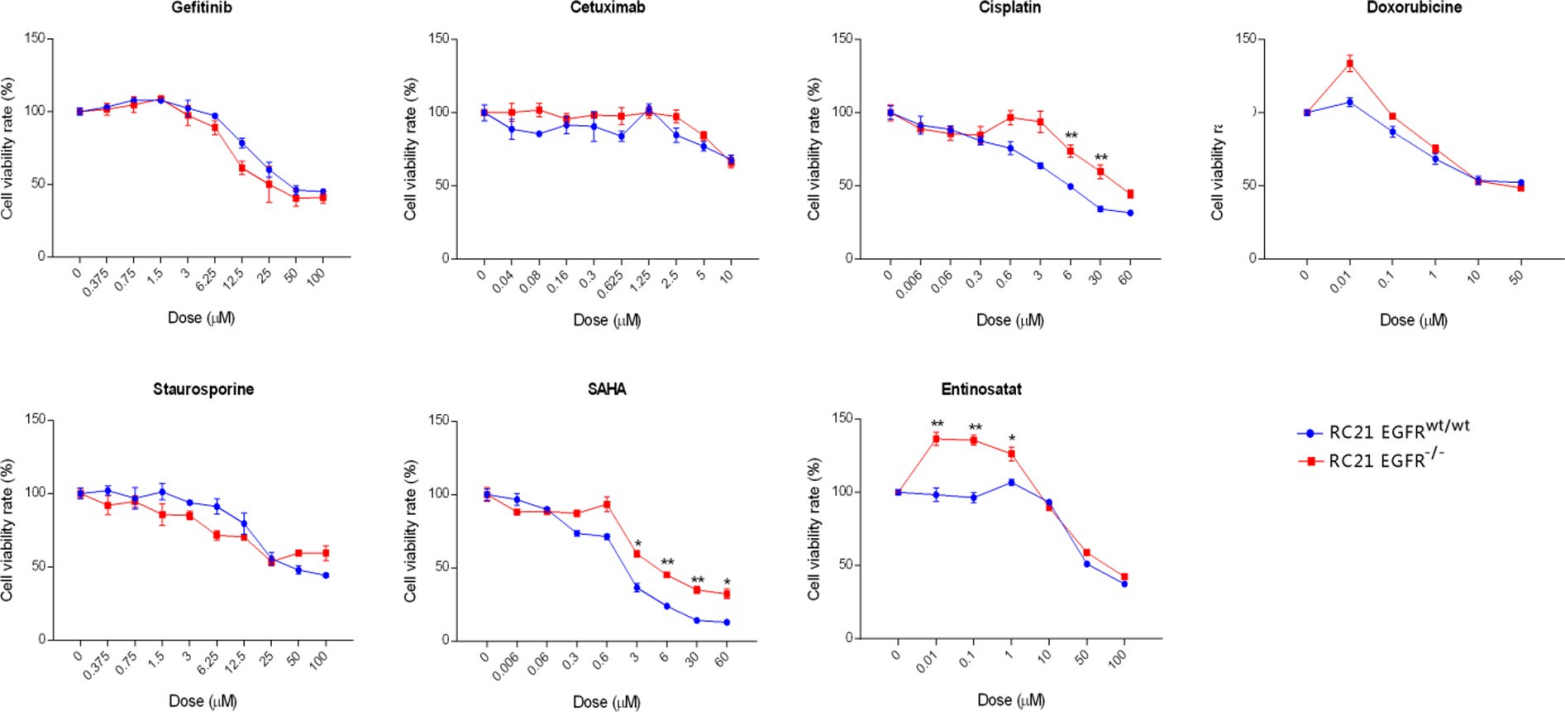
Treatment of the RC21 *EGFR*^*wt/wt*^ and *EGFR*^*-/-*^ cells with different anti-cancer drugs. Cells were treated with the indicated drugs at indicated concentrations for 72 hrs and the cell viability was determined using MTS assay. For staurosporine, cell viability was measured after 24 hrs due to its toxicity. All data in the graphs are represented as mean ± SD (n≥3), two-tailed unpaired student’s t-test: *p-values <0.05; **p-values <0.01; ***p-values <0.001.

To characterize the effect of EGFR loss on cancer cell growth, a clonogenic assay was performed. RC21 *EGFR*^-/-^ cells showed a significant reduction in cell proliferation and colony formation property (Fig 2). Then, we tested several targeted and chemotherapeutic drugs on RC21 *EGFR*^*wt/wt*^ and *EGFR*^*-/-*^ cells. Interestingly, RC21 *EGFR*^-/-^ cells showed resistance to cisplatin and SAHA in a dose dependent manner. We did not observe significant differences between RC21 *EGFR*^*wt/wt*^ and *EGFR*^*-/-*^ cells upon treatment with TKIs and cetuximab (Fig 3).

### EGFR loss leads to ERK activation

**Fig 4:**
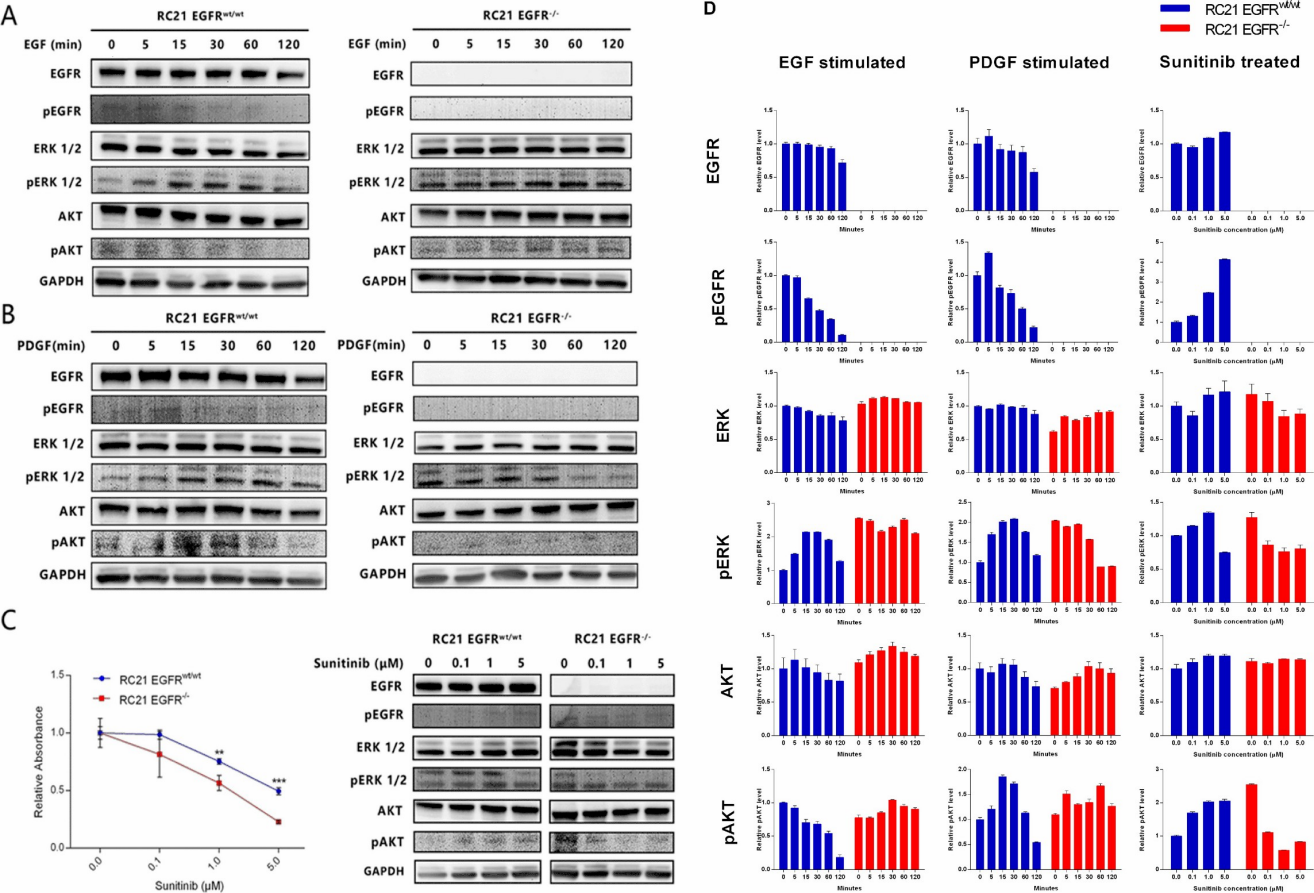
Downstream pathways and cell proliferation alterations with different treatments (A) Western blot analysis of RC21 *EGFR*^*wt/wt*^ and *EGFR*^*-/-*^ cells stimulated with EGF at different dose and time points. (B) Western blot analysis of RC21 *EGFR*^*wt/wt*^ and *EGFR*^*-/-*^ cells stimulated with PDGF at different dose and time points. (C) Clonogenic assay to test cell proliferation and western blot analysis of downstream pathway after treatment with sunitinib. (D) Western blot quantification. The graph was generated through quantifying blots from three independent experiments by ImageJ and normalizing the intensity of the bands to the lane at 0 min.

To investigate the impact of EGFR loss on the key downstream signaling pathways, we assessed alterations in the expression of MAPK/ERK and PI3K/Akt in RC21 *EGFR*^*wt/wt*^ and *EGFR*^*-/-*^ cells. We specifically evaluated expression of EGFR, pEGFR, Akt, pAkt, ERK and MAPK (pERK) by Western blot. As expected, we did not detect EGFR or pEGFR in *EGFR*^*-/-*^ cells (Fig 4A and 4D). However, we observed a relatively higher level of pERK1/2 in RC21 *EGFR*^*-/-*^ cells as compared to *EGFR*^*wt/wt*^ cells with or without stimulation with EGF or PDGF (Fig 4A and 4B). We also found a lower level of pAKT in RC21 *EGFR*^*-/-*^ cells than the parental cells upon stimulation with PDGF (Fig 4B).

### Sunitinib attenuates pERK1/2 and pAKT levels and further inhibits RC21 *EGFR*^*-/-*^ cell proliferation

Sunitinib is a receptor TKI that can inhibit cellular signaling induced by VEGFR and PDGFR. To determine the effect of sunitinib on cell proliferation and downstream pathway, we treated RC21 *EGFR*^*wt/wt*^ and *EGFR*^*-/-*^ cells with sunitinib at different doses. We observed that sunitinib induces a marked suppression of proliferation in RC21 *EGFR*^*-/-*^ cells as compared to RC21 *EGFR*^*wt/wt*^. Furthermore, we found that the highly expressed pERK1/2 and pAkt introduced by EGFR loss can be abolished by sunitinib in *EGFR*^*-/-*^ cells (Fig 4C and 4D).

### EGFR loss reduces G0/G1 phase population and leads to resistance to apoptosis

**Fig 5:**
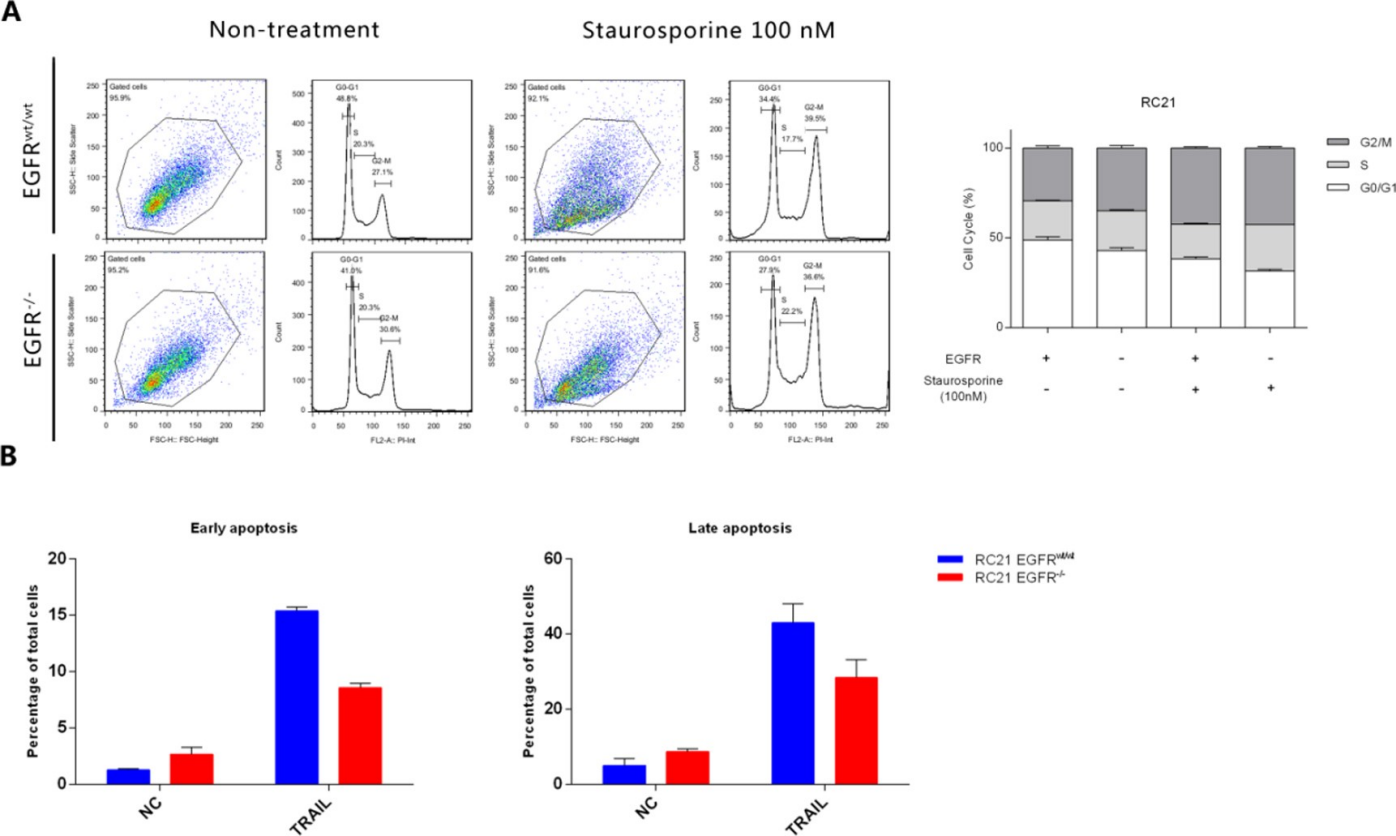
Annexin-V/Propidium Iodide staining for cell cycle and apoptosis analysis of RC21 *EGFR*^*wt/wt*^ and *EGFR*^*-/-*^ cells. (A) Propidium Iodide staining for cell cycle analysis in the presence and absence of Staurosporine. (B) Annexin-V/Propidium Iodide staining for apoptosis analysis in the presence and absence of TRAIL.

To determine the effect of EGFR loss on cell cycle and apoptosis, an APC-conjugated Annexin-V/Propidium Iodide assay was performed. Loss of EGFR decreased G0/G1 phase population as and induced G2/M phase arrest. Moreover, G0/G1 phase population in RC21 *EGFR*^*-/-*^ cells was further decreased upon treatment with staurosporine (100 nM) (Fig 5A). For apoptosis analysis, we used trimeric recombinant human TRAIL (rhTRAIL) to stimulate the apoptotic pathway. Our data show that EGFR loss leads to resistance to apoptosis induced by rhTRAIL (50ng/ml) in renal cancer cells (Fig 5B).

## Discussion

EGFR plays an essential role in the tumorigenesis of a variety of cancers including RCC, where it is commonly overexpressed. EGFR expression is considered as an important biomarker for predicting response to TKIs. Nevertheless, so far, no satisfactory therapeutic results have been achieved using EGFR-TKIs in clinical trials for RCC. In this study, we examined whether *EGFR* knockout in combination with different small molecular inhibitors or a therapeutic protein (TRAIL) can be used as a treatment option for RCC. We showed that disruption of overexpressed EGFR dramatically inhibits the proliferation of RCC and arrests cells at G2/M checkpoint. Furthermore, we found that inhibition of PDGFR and VEGFR by sunitinib can attenuate the expression of pERK1/2 and pAKT induced by EGFR loss. We did not observe any difference in the viability of RC21 *EGFR*^*wt/wt*^ and *EGFR*^*-/-*^ cells after gefitinib treatment. This can be explained by the significantly higher affinity of gefitinib to the *EGFR* mutant cells than to the wild-type.

Overexpression of EGFR is associated with poorer survival in many cancers [14,15]. We showed that RC21 also has a much higher expression of EGFR as compared to a number of commonly used cell lines, such as HEK293, DLD1, A549 and Hela cells. Of note, human embryonic kidney cells (HEK293) have the lowest expression levels of EGFR among the tested cell lines as compared to RC21, suggesting EGFR as a tumor biomarker or target in RCC. Overexpression of EGFR is thought to play an important role in proliferation and survival of tumor cells in a variety of cancers [16]. It is also considered as a response biomarker for EGFR-TKIs or EGFR-antibodies, including gefitinib, elortinib, afatinib and cetuximab [17].

The underlying mechanisms of limited drug response and resistance to EGFR targeted therapies in RCC are not fully understood which is mainly due to lack of means for complete elimination of EGFR from cells. It is generally considered that the RNAi and shRNA cannot efficiently inhibit EGFR expression and the residual EGFR in cells may contribute to tumor development [18]. Besides, homozygous *EGFR* knockout in mice results in an early embryonic lethality [16]. In addition, EGFR inhibitors cannot completely inhibit EGFR signaling because of dose limitations toxicity and others. EGFR inhibitor (tyrosine kinase inhibitor) could block the tyrosine kinase activity of EGFR, but it cannot fully block other function of EGFR. It has been reported that EGFR has various functions other than tyrosine kinase activity, such as many ligands depending functions, crosstalk with other proteins and others [19,20]. Thus, the precise role of EGFR in tumor development is difficult to unravel. Here, we generated RC21 *EGFR* knockout cell line by HDR using CRISPR/Cas9. We show that EGFR loss inhibits renal cancer cell proliferation. It indicates CRISPR-mediated disruption of *EGFR* may be a promising therapeutic option for RCC in the future [21]. Given the importance of EGFR overexpression for tumor survival, growth and drug resistance, future studies are needed to explore whether overexpressed *EGFR* knockout can be an option for more cancers [22]. However, for clinical use, optimization of the delivery methods for specifically targeting overexpressed EGFR in cancer cells needs more in depth investigations [23]; for instance, optimization of specific gene therapy delivery vehicles based on EGFR [24]

We showed a higher level of MAPK/pERK in RC21 *EGFR*^*-/-*^ cells as compared to the *EGFR*^*wt/wt*^ cells indicative of a bypass mechanism for activation of MAPK/pERK pathway upon loss of EGFR. We observed that the proliferation of RC21 *EGFR*^*-/-*^ can be inhibited by sunitinib. Furthermore, the overactivated MAPK/pERK and pAKT in RC21 *EGFR*^*-/-*^ cells are inhibited by sunitinib suggesting VEGFR and/or PDGFR may be implicated in this bypass mechanism. However, a phase I/II trial did not show sunitinib plus gefitinib more efficacy to sunitinib monotherapy [5]. One possibility might be that gefitinib only shows therapeutic effects on patients with certain *EGFR* mutations, however, in this clinical trial, it was unknown whether these RCC patients had *EGFR* mutations or not. In concordance with our data, several studies have shown that reactivation of MAPK/ERK signaling pathway frequently occurs in TKIs-based therapies [25–28]. One of the most common adverse events for treatment of RCC is hypertension, which is closely correlated to the dose of VEGFR inhibitors (sunitinib, etc.) [29]. Future studies are needed to further investigate the outcomes of hypertension upon the combination of EGFR and/or VEGFR/PDGFR inhibition. Altogether, evaluation of certain receptor tyrosine kinases before and after treatment could be beneficial for patients with RCC. Furthermore, combination targeted therapy might be a more promising strategy to overcome drug resistance in these patients.

Our results show that EGFR loss leads to the resistance of renal cancer cells to cisplatin, HDAC inhibitors and TRAIL [30–32]. Several studies have shown that the EGFR status is associated with drug resistance in cancer [33–35]. According to our observations, disruption of overexpressed EGFR suppresses cancer cell growth, but ultimately leads to the reactivation of pERK and/or pAKT via an EGFR independent mechanism and drug resistance. We previously reported a promising anticancer activity of EGFR-Selective TRAIL Fusion Protein in RC21 [11]. However, in this study we show that loss of EGFR results in the resistance of cancer cells to TRAIL. Future studies should include more RCC with different subtypes, and those results observed in vitro should be further confirmed by *in vivo* studies before moving to clinic. Also, it would be important to check the patients *EGFR* status (mutations or overexpression) before treatment if EGFR inhibitors or antibodies are applied in clinic. Taken together, the response of tumor cells to TRAIL might also be related to the expression level of EGFR.

**Fig 6:**
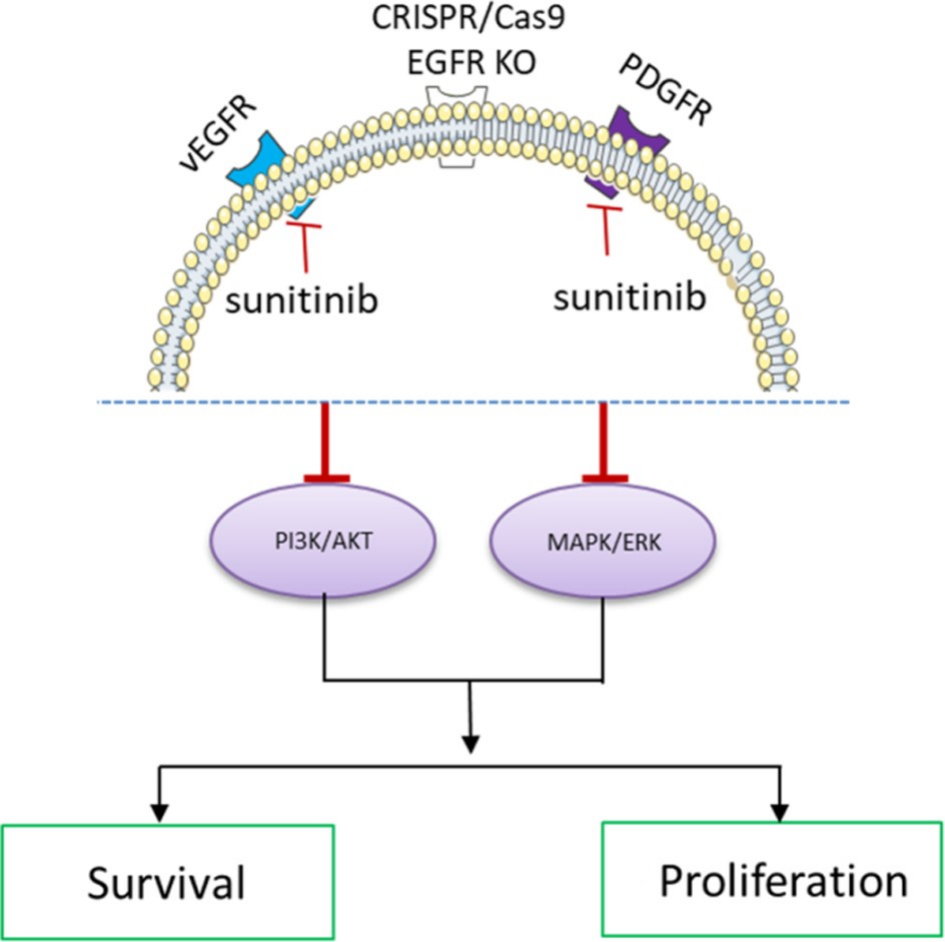
Proposed model of CRISPR/Cas9 mediated EGFR knockout in combination with sunitinib and its effect on MAPK signaling pathway in renal cell carcinoma.

In conclusion, knockout of overexpressed EGFR dramatically inhibits renal cancer cell growth. Although EGFR loss leads to cell survival and multiple drug resistance, sunitinib can further inhibit renal cancer cell proliferation upon loss of EGFR (Fig 6).

## Supporting information

S1 Fig Flow cytometric analysis of EGFR in RC21, Hela, A549 and DLD1 and HEK293 cells. (TIF)

S2 Fig Targeted gene disruption was pre-assessed by using the T7EI-based assay in HEK293 cells. (TIF)

S1 Data (JPG)
