# Supplementary figures and images for: CRISPR-mediated ablation of overexpressed EGFR in combination with sunitinib significantly suppresses renal cell carcinoma proliferation
Source: PLoS One. 2020 May 15;15(5):e0232985. doi: 10.1371/journal.pone.0232985 (PMC7228069; doi:10.1371/journal.pone.0232985)

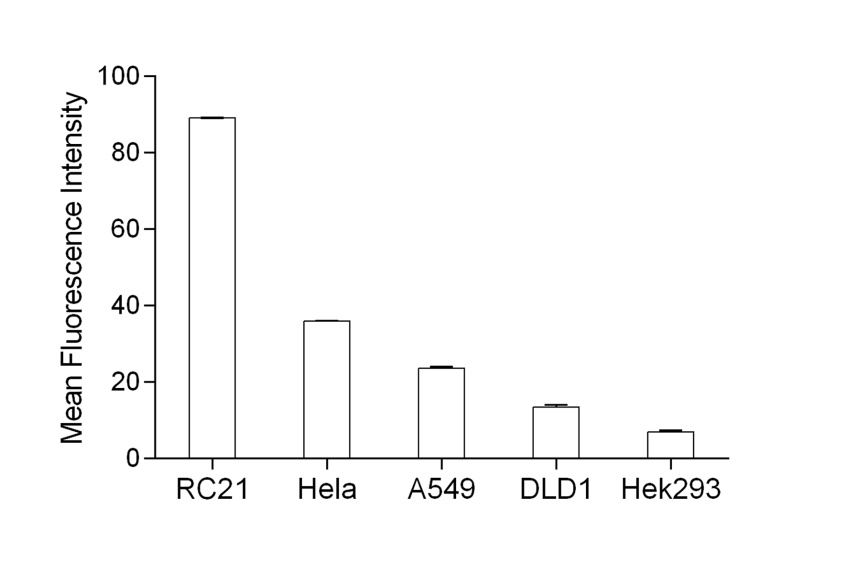

Supplement: S1 Fig — (TIF) [file pone.0232985.s001.tif]

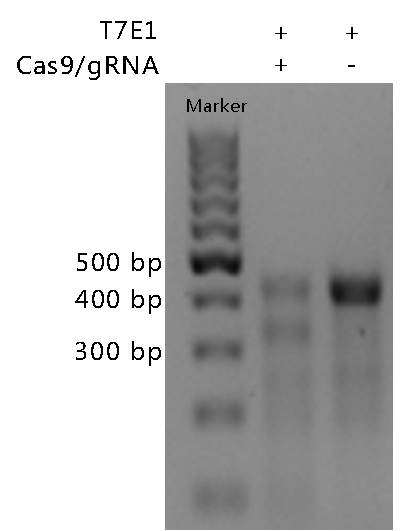

Supplement: S2 Fig — (TIF) [file pone.0232985.s002.tif]

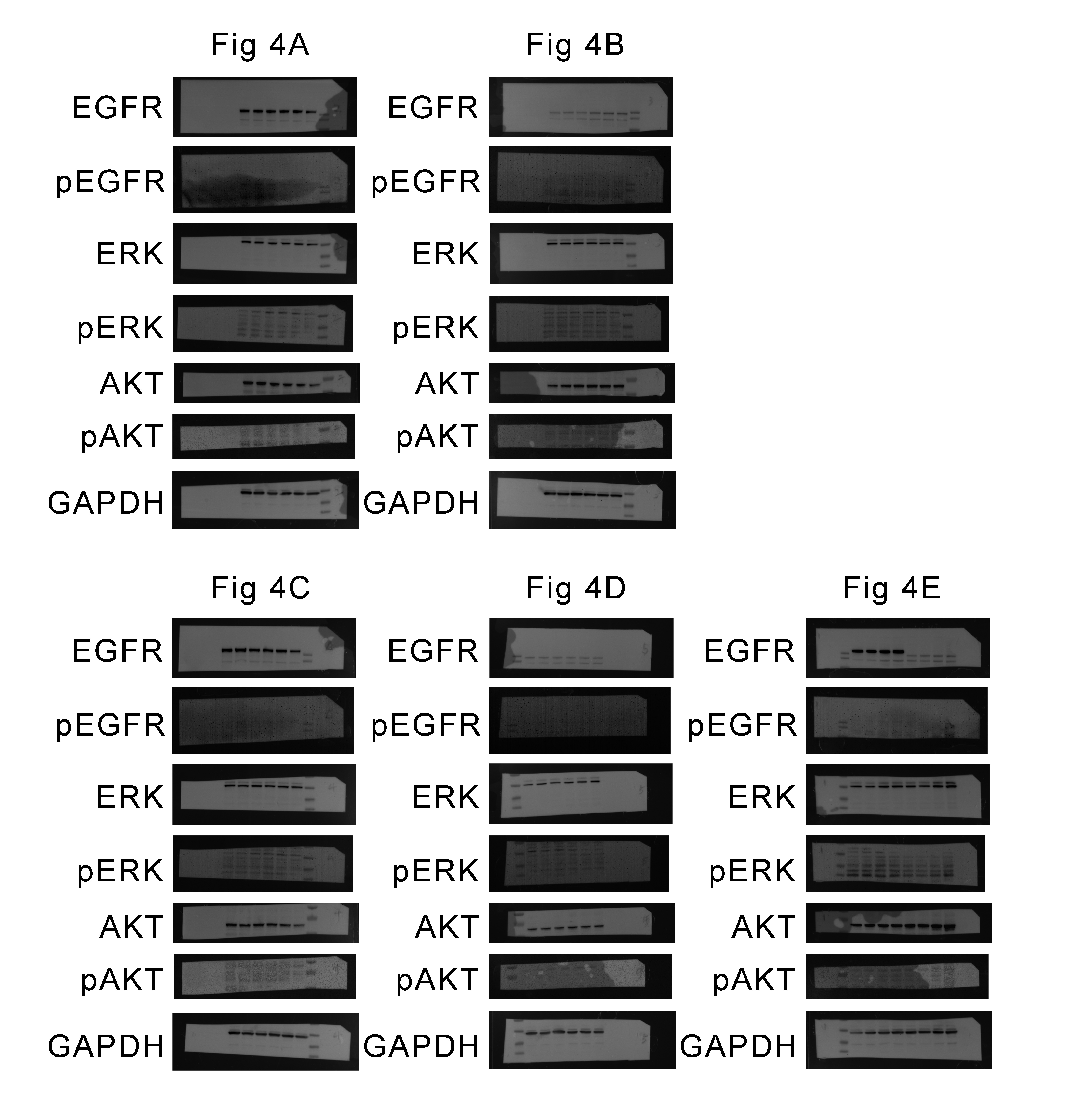

Supplement: S1 Data — (JPG) [file pone.0232985.s003.jpg]
